# Design and Validation of Equiaxial Mechanical Strain Platform, EQUicycler, for 3D Tissue Engineered Constructs

**DOI:** 10.1155/2017/3609703

**Published:** 2017-01-12

**Authors:** Mostafa Elsaadany, Matthew Harris, Eda Yildirim-Ayan

**Affiliations:** ^1^Department of Bioengineering, University of Toledo, Toledo, OH, USA; ^2^Department of Orthopaedic Surgery, University of Toledo Medical Center, Toledo, OH, USA

## Abstract

It is crucial to replicate the micromechanical milieu of native tissues to achieve efficacious tissue engineering and regenerative therapy. In this study, we introduced an innovative loading platform, EQUicycler, that utilizes a simple, yet effective, and well-controlled mechanism to apply physiologically relevant homogenous mechanical equiaxial strain on three-dimensional cell-embedded tissue scaffolds. The design of EQUicycler ensured elimination of gripping effects through the use of biologically compatible silicone posts for direct transfer of the mechanical load to the scaffolds. Finite Element Modeling (FEM) was created to understand and to quantify how much applied global strain was transferred from the loading mechanism to the tissue constructs. In vitro studies were conducted on various cell lines associated with tissues exposed to equiaxial mechanical loading in their native environment. In vitro results demonstrated that EQUicycler was effective in maintaining and promoting the viability of different musculoskeletal cell lines and upregulating early differentiation of osteoprogenitor cells. By utilizing EQUicycler, collagen fibers of the constructs were actively remodeled. Residing cells within the collagen construct elongated and aligned with strain direction upon mechanical loading. EQUicycler can provide an efficient and cost-effective tool to conduct mechanistic studies for tissue engineered constructs designed for tissue systems under mechanical loading in vivo.

## 1. Introduction

The interactions between cells and their microenvironment play a crucial role in driving cellular and molecular changes towards proliferation, migration, apoptosis, and differentiation. Among these interactions, the mechanical forces around the cells comprise an important facet of cellular hemostasis [1–3]. Primarily, animal models have been used in studying these interactions [4]; however, in vivo studies are associated with limited reproducibility, prohibitive cost, and difficulty in data interpretation due to synergetic effects of multivariable factors [5]. As a result, physiologically relevant three-dimensional (3D) in vitro platforms have been developed to understand the role of exogenous mechanical forces in cellular functions.

In last ten years, in vitro mechanical loading platforms have been essential in studying the solo effect of mechanical forces or force-induced strains on cells [6]. These platforms have the specific aim of applying adjustable static or cyclic predefined strains and frequency to the cells or cellularized constructs. They apply tension and compression using uniaxial, biaxial, and equiaxial loading modalities to 3D cell-embedded constructs to recapitulate key aspects of in vivo mechanical environment niches [7–10]. The choice of the mechanical loading modalities is dependent on which tissue is being studied and what types of mechanical loading that tissue experiences in its physiological state.

Innovative and versatile mechanical loading platforms have been introduced to the literature, and some were commercialized [11–14]. One of the critical issues in most of these mechanical platforms is the creation of nonuniform stress distribution on the mechanically loaded constructs. These platforms commonly employ various gripping or clamping systems to hold the cellularized construct either from one end of the constructs or from both ends to apply the mechanical strains. As a consequence, this creates local disturbance in stress pattern and generates higher stress concentrations in the immediate vicinity of gripped area compared to the rest of the construct [15]. This suggests that cells loaded with these systems do not receive uniform mechanical strain and mechanical signals within the 3D construct [15–17]. As known from the literature, cells are very sensitive to the mechanical stress around them [17, 18], which in fact control deformation and differentiation status of the cells [19]. Thus, there is a great demand for a mechanical loading platform, which can apply homogenous mechanical strains to 3D cellularized construct without using any gripping apparatus or fixtures [16].

In this study, we aimed (i) to introduce an innovative mechanical loading platform called EQUicycler to the literature that is able to apply equiaxial mechanical strain homogenously to 3D cell-embedded collagen construct without creating griping effects, (ii) to evaluate the strain transfer performance of EQUicycler using computational modeling, and (iii) to evaluate the feasibility of utilizing EQUicycler to support the viability of musculoskeletal tissue related cells and to evaluate the subsequent changes in cell and matrix morphology. The results show that EQUicycler promotes collagen fiber alignment, encapsulated cell alignment, and cell viability throughout 3D collagen construct.

## 2. Materials and Methods

### 2.1. Design of Innovative Mechanical Loading Platform of 3D Cell-Embedded Constructs: EQUicycler

**Figure 1:**
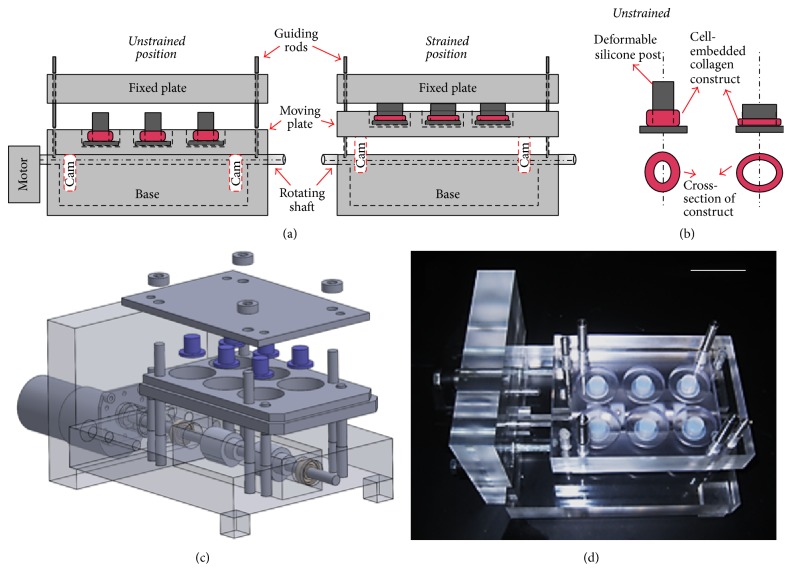
Schematic and optical images of EQUicycler. (a) Schematic representation of EQUicycler system with its major components and position of the moving plate and silicone posts prior to and during the mechanical loading. (b) Schematic view of cell-embedded collagen construct around silicone post prior to and during the mechanical loading. (c) Three-dimensional solid model and exploded diagram of EQUicycler. (d) Optical images of EQUicycler. Scale bar represents 1 inch.

The EQUicycler, an innovative custom-built mechanical loading platform, is created to apply cyclic equiaxial mechanical strain with predefined frequency to the cells-embedded 3D collagen constructs. The EQUicycler system consists of four major components: (1) a pear-shaped cam mechanism containing a rotating shaft and two cams; (2) a moving plate hosting deformable silicone posts; (3) deformable silicone posts hosting cell-embedded collagen matrix around it, and (4) a motor mechanism rotating the shaft with predefined frequency.  Figure 1 shows the schematic and optical image of EQUicycler system with its major components and schematic of silicone posts with cell-embedded collagen matrix prior to and during the mechanical loading.

The EQUicycler's working mechanism is based on creating a mechanical strain on silicone post hosting cell-embedded collagen ring around it. The motor with adjustable frequency rotates the shaft with pear-shaped cams. As the cams rotate, the moving plate, which is placed on the cams, moves up and down. The displacement distance of the moving plate is defined by the geometry of the cams. During this reciprocating motion, the displacement of the moving plate against the fixed plate causes compression of the silicone post. This compression further creates mechanical strain in the cell-embedded collagen ring, which surrounds the deformable silicone post.

The EQUicycler eliminates the problems associated with the gripping effects as experienced in the majority of mechanical loading platforms. The grips used at either end of the cell-embedded construct to mount the construct to the platforms [20–25] create stress/strain spike in the vicinity of the gripped area that leads to a nonhomogenous stress/strain distribution compared to the areas in the middle of the sample [26–28]. The EQUicycler is designed such that the cell-embedded collagen construct is integrated into the platform by depositing it around a deformable silicone post rather than using grips. The compression of the silicone post produces mechanical strain in the cell-embedded collagen ring, which surrounds the post. Therefore, homogeneous strain throughout the cell-embedded collagen construct can be created without creating a stress concentration.

In the EQUicycler system, the applied mechanical strain and its profile are dictated by the geometry of cam mechanism. In the pear-shaped cam system, during rotation, cam remains motionless, and the moving plate becomes stationary for two-thirds of a revolution. This period is called a dwell period. During the other third of the revolution of the cam, the moving plate rises and then falls. As the pear-shaped cam is symmetrical, the rise motion is the same as the fall motion. This profile creates cyclic displacement for the moving plate, which is utilized in the EQUicycler system to create cyclic reciprocating motion for the moving plate.

**Figure 2:**
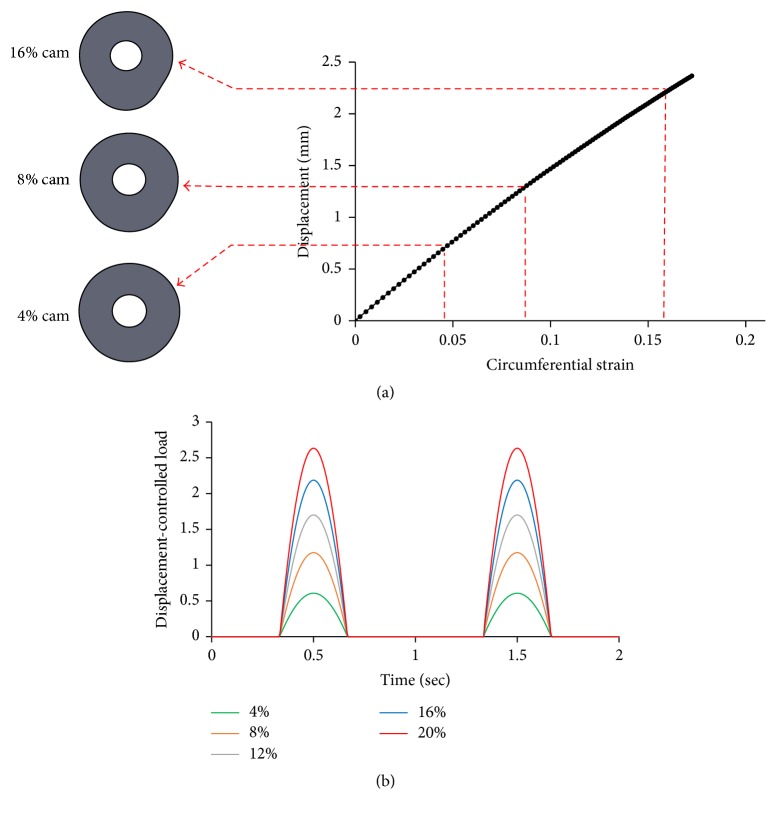
(a) Cam profiles used to create 4%, 8%, and 16% equiaxial mechanical strains and the correlation between the displacement of moving plate and applied circumferential strain. (b) Displacement-controlled load versus time graph with representative loading-unloading cycles for various equiaxial mechanical strains.

Finite Element Modeling (FEM) was used to design the cam mechanism. The displacement of the silicone post was correlated with the maximum circumferential strain on the collagen constructs.  Figure 2 demonstrates the correlation between the displacement of moving plate and circumferential strain and representative profile of two loading-unloading cycles for various equiaxial mechanical strains generated using EQUicycler.

### 2.2. Investigating Strain Transfer to the 3D Cell-Embedded Collagen Construct in EQUicycler: FEA Analysis

**Figure 3:**
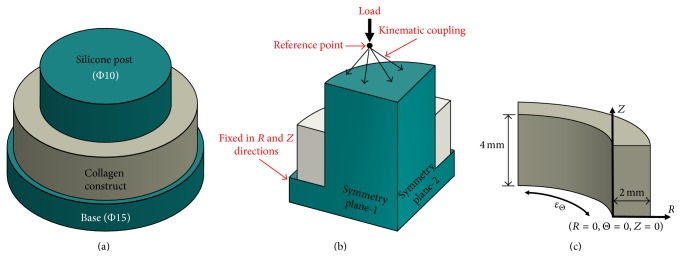
(a) Schematic view of the silicone post and collagen constructs assembly. (b) A quarter model that depicts the two planes of symmetry used in the FE model and the associated loading and boundary conditions. (c) Representation of the collagen part of the model with the associated local coordinate system used in the postprocessing of the model results. *R*, *θ*, and *Z* are radial, circumferential, and longitudinal coordinates.

Finite Element Analysis (FEA) was utilized to investigate how applied mechanical strain is transferred to the 3D collagen construct and to understand whether there is any strain attenuation in radial and longitudinal direction upon mechanical loading. FE models were developed and solved using ABAQUS (Dassault Systems, USA). The silicone post and cell-embedded collagen constructs were simulated as homogeneous isotropic materials. The schematic views of silicone post and collagen construct are given in Figure 3.

*Geometry and Meshing.* The silicone post has a cylindrical base of 15 mm diameter and 2 mm height while its protrusion is 10 mm in diameter and 8 mm in height. The collagen construct was modeled as an annular ring with 10 mm inner and 14 mm outer diameters and 4 mm height. Two planes of symmetries were defined in the model. Hence, only one-quarter of both silicone and collagen was modeled to improve the computational efficiency. The geometry of the silicone posts and the collagen rings that was used in FEM is shown in Figure 3. Both silicone and collagen parts were meshed with C3D8H 8-node linear brick, hybrid, and constant pressure elements to allow for the nonlinear deformations of the model materials. Mesh convergence study was performed, and the silicone part of the model was meshed with 5616 elements and collagen part was meshed with 2880 elements.

*Material Properties.* Ogden hyperelastic material model was used to define the mechanical properties of the silicone posts. The Ogden material parameters were obtained by performing compression testing of the silicone posts. The resulting stress-stretch curves from mechanical testing were fitted to Ogden model [29, 30]. Collagen viscoelastic time-dependent material properties were modeled using Prony series. Stress relaxation data was obtained from Pryse et al. [31] and fitted to Prony series model to obtain the material shear moduli and time constants.

*Loading and Boundary Conditions.* The displacement-controlled load is applied monotonically to a reference point that is kinematically coupled to the upper surface of the silicone post. The reference point that was used to apply the load was fixed in all the degrees of freedom except the longitudinal direction. The bottom surface of the silicone post base is fixed in the longitudinal direction, and lateral surface is fixed in the radial direction. Frictionless surface-to-surface contact assumption was imposed at the silicone post and collagen rings interface to simulate the experimental setup in which the samples are submerged in cell culture media. During loading, cell culture media act as a lubricant in the silicone-collagen interface. The applied loading and boundary conditions are shown in Figure 3.

*Postprocessing.* Cylindrical coordinates are described in a normalized form as *R* and *Z*, where *R* is the normalized radial and *Z* is the normalized longitudinal coordinate. *R* is obtained by dividing the radial coordinate (*r*) by the outer radius of the collagen construct (or, 14 mm). *Z* is obtained by dividing the *z*-coordinate by the total height of the collagen construct (4 mm). Percentage strain attenuation is calculated by dividing the difference between the maximum and minimum strain in the model by the maximum strain. From FEA, we demonstrated how applied strain is transferred in longitudinal and radial direction within the collagen construct.

### 2.3. Performance Analyses of EQUicycler

#### 2.3.1. Preparing and Mechanical Loading of Cell-Embedded Collagen Construct

Prior to mechanical loading, cell-embedded collagen scaffolds were prepared. Skeletal muscle cells (C2C12) and osteoprogenitor cells (MC3T3-E1) were utilized to analyze EQUicycler's capacity with different cell lines. C2C12 (ATCC, USA) cells were cultured in Dulbecco's Low-Glucose Modified Eagle's Medium (DMEM) (ThermoFisher, USA) supplemented with 10% Fetal Bovine Serum (FBS) (Gibco, USA) and 1% penicillin/streptomycin (Life Technologies, USA). MC3T3-E1 (ATCC, USA) were maintained in complete media comprising Alpha-Minimum Essential Medium (*α*-MEM) (Life Technologies, USA) supplemented with 10% FBS (Gibco, USA) and 1% penicillin/streptomycin (Life Technologies, USA). Upon confluence, cells were harvested and encapsulated within the collagen type-I solution with 10^6^ cells/mL seeding density. For the cellular study, 2.5 mg/mL collagen type-I (Corning, USA) solution was prepared from stock collagen type-I solution. Briefly, collagen type-I stock solution at 3.7 mg/mL concentration with pH ~ 3-4 was diluted to 2.5 mg/mL and neutralized with chilled 1 N NaOH, phosphate buffer saline (PBS), and deionized water according to manufacturer's protocol. C2C12 cells or MC3T3-E1 cells-embedded collagen constructs were then deposited around the silicone posts and incubated overnight. Next day, scaffolds were exposed to various equiaxial strains (4% and 8%) with 1 Hz frequency using EQUicycler. Unstrained (0%) scaffolds were used as control group.

#### 2.3.2. Assessing Cell Proliferation, Viability, and Differentiation

The proliferation of the encapsulated cells in collagen was examined using nondestructive alamarBlue (aB) assay (Biosource International, USA). Four samples (*n* = 4) were used for each of the characterization and control groups. On the day of characterization, the samples were incubated with 10% (v/v) of aB assay solution in cell culture media for 6 hours. After the incubation period, the reduced color of the cell culture media was quantified by measuring the fluorescence intensity using a microplate fluorometer (Wallac 1420, USA) at 545/585 nm excitation/emission wavelengths.

The viability of the encapsulated cells at day 7 was assessed using live/dead assay (Life Technologies, USA). Upon incubation with live/dead assay reagents, confocal images of live and dead cells within 3D scaffolds were obtained. On the characterization day, the samples were first rinsed twice with PBS for 5 min and then incubated for 30 minutes with a 1 : 4 concentration ratio of Calcein-AM for labeling live cells (green) and ethidium homodimer-1 for labeling dead cells (red). After incubation, the samples were fixed using 4% paraformaldehyde (Sigma-Aldrich, USA) in PBS for 30 minutes and washed thrice with PBS for 15 minutes and stored at 4°C until they were imaged using a Leica TCS SPE confocal microscope. Cells stained with Calcein-AM were visualized using a 488 nm excitation and an emission spectrum of 491 nm to 545 nm wavelength. Cells stained with ethidium homodimer-1 were visualized using a 568 nm excitation and an emission spectrum of 590 nm to 685 nm wavelength.

The cells alkaline phosphatase (ALP) activity was evaluated at day 14 and day 21 using ALP colorimetric assay (Abcam, USA). Before conducting the assay, the encapsulated cells within the collagen scaffolds were liberated using 0.2% (w/v) collagenase type-I solution (Life Technologies, USA) in complete media. The samples were incubated in the collagenase solution for 1 h at 37°C in shaking water bath. After collagenase digestion, the solution was centrifuged to isolate the cell pellet. The ALP assay was performed based on the manufacturer's protocol (Abcam, USA). Briefly, the amount of p-nitrophenol (pNP) converted from p-nitrophenyl phosphate (pNPP) substrate in the presence of ALP was quantified by measuring the absorbance at 405 nm using a microplate reader (SOFTmax Pro). A calibration curve obtained using a serial dilution of pNP was used to convert the measured absorbance to pNP value.

#### 2.3.3. Changes in Collagen Fiber Morphology and Alignment

The morphological changes on 3D collagen scaffold were examined by Scanning Electron Microscopy (SEM) (Quanta 3D FEG) and histological analyses. For SEM examination, first, control and mechanically strained collagen constructs were fixed with 4% paraformaldehyde solution (Sigma-Aldrich, USA) for 1 h. In order to preserve the collagen fibrils and improve the image quality the samples were, then, postfixed in 1% (v/v) osmium tetroxide (Across Organics, USA) diluted with Sorensen's buffer. After fixation, the samples were washed twice with PBS and subsequently dehydrated in increasing concentrations of ethanol from 30% to 100% and ethanol/hexamethyldisilazane (HMDS) (Fisher Scientific, USA) solutions from 30% to 100% for 15 min each. Additionally, the samples were air-dried overnight to evaporate the residual organic solvents. Before SEM examination, the dried samples were gold sputter-coated to avoid charge accumulation.

The changes in collagen fiber morphology and alignment in mechanically strained samples compared to control were visualized using histological analysis and Mason's Trichrome staining. On the characterization day, cell-encapsulated constructs were fixed in 10% formalin for 24 h, dehydrated using an ethanol gradient, and incubated in xylene before embedding them in paraffin. The embedded samples were sectioned at 20 *μ*m thickness and mounted on microscope slides. The rehydrated sections were incubated in Mason's Trichrome stain, washed, and viewed under a bright field microscope to observe collagen fibers and cells.

### 2.4. Statistical Analysis

One-way Analysis of Variance tests were performed using SPSS Statistics Package (IBM, USA) to determine if there is a significant difference between the means of the experimental and control groups. Fisher's LSD post hoc was used for multiple comparisons between different groups. Statistical significance was defined as *p* < 0.05.

## 3. Results

### 3.1. Finite Element Analysis (FEA) of the Strain Transfer in the Collagen Constructs

**Figure 4:**
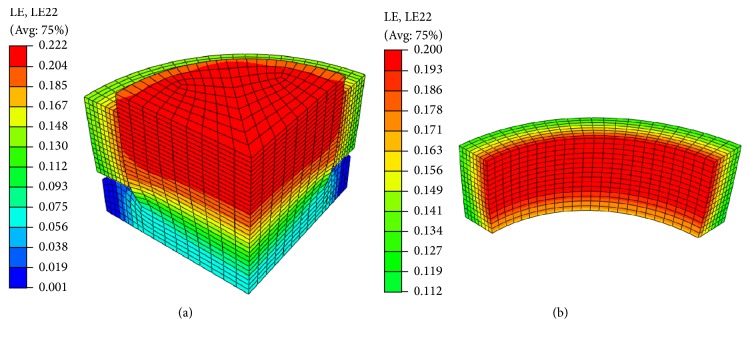
Circumferential strain contour of (a) the silicone-collagen assembly and (b) the collagen construct only. Representative global 20% strain loading is shown.

Representative circumferential strain contours for the silicone-collagen assembly and the collagen construct alone are shown in Figure 4. The displacement-controlled loading pertinent to the cam profiles utilized in EQUicycler resulted in the compression of silicone posts in the longitudinal direction and expansion in the circumferential direction (Figure 4(a)). Circumferential strain in the silicone posts was transferred to the collagen rings through the nonlinear surface-to-surface interaction imposed at the silicone-collagen interface. The circumferential strain contours of collagen construct in Figure 4(b) show that the distribution of the strain is similar to the longitudinal direction.

**Figure 5:**
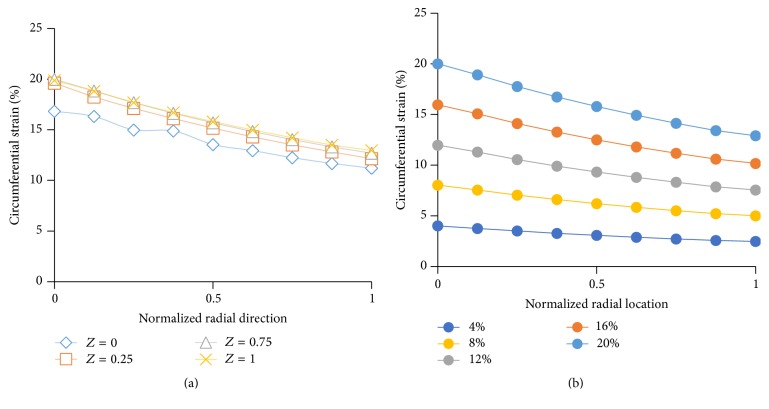
(a) Representative circumferential strain gradients along the normalized radial direction of the collagen matrix for four different longitudinal positions. Results are reported at *θ* = 0 under 20% strain loading. (b) The average circumferential strain gradient in the collagen construct along the radial direction of the different mechanical strain.

The circumferential strain gradients in normalized radial direction for different longitudinal coordinates (Figure 5(a)) and for different mechanical strains (Figure 5(b)) were shown in Figure 5. As seen in Figure 5(a), the strain gradients over the radial direction for different longitudinal coordinates (*Z*) were almost identical to four different positions with the exception of *Z* = 0. Moreover, the circumferential strain gradients in normalized radial direction were obtained at different loading conditions (Figure 5(b)). The strain attenuation increased slightly with increased mechanical strain. As a representative example, for normalized radial direction at 1 (at the outer region of the collagen construct), the strain attenuation was 35.5% for 4% strain and was 38.4% for 20% strain.

### 3.2. Effect of Mechanical Loading on Cells and Collagen Matrix

**Figure 6:**
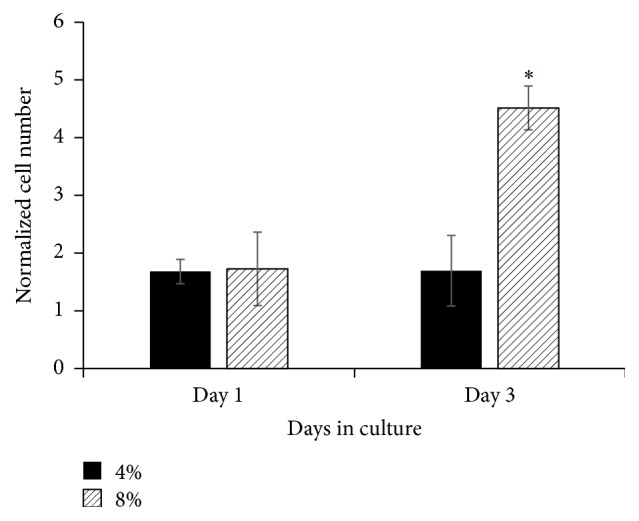
Normalized C2C12 cell number within 4% and 8% mechanically strained collagen. Normalized cell number is the ratio of cell number in mechanical strain group over cell number in the control group on culture day. *∗* indicates significant difference from other groups.

*Cell Viability.* C2C12 cell-encapsulated within the collagen matrix was exposed to 4% and 8% equiaxial strains with 1 Hz frequency for three days with 3 hours/day loading. These magnitudes of mechanical strains were chosen based on physiological loading environment of muscle cells [32–34]. Unstrained cell-embedded collagen served as a control.  Figure 6 demonstrates the changes in normalized C2C12 cell number with increased equiaxial strain. Muscle cell viability data demonstrates that equiaxial strains applied using EQUicycler did not decrease the cell viability. On day 1, the normalized cell number was not statistically different between 4% and 8% strained cells. On day 3, however, the normalized cell numbers were higher for 8% strained cells compared to those exposed to 4% equiaxial strain. Increased strain magnitude increased the normalized cell number in EQUicycler system. For C2C12 cells, mechanical loading using EQUicycler positively affected the cell viability during the culture period.

**Figure 7:**
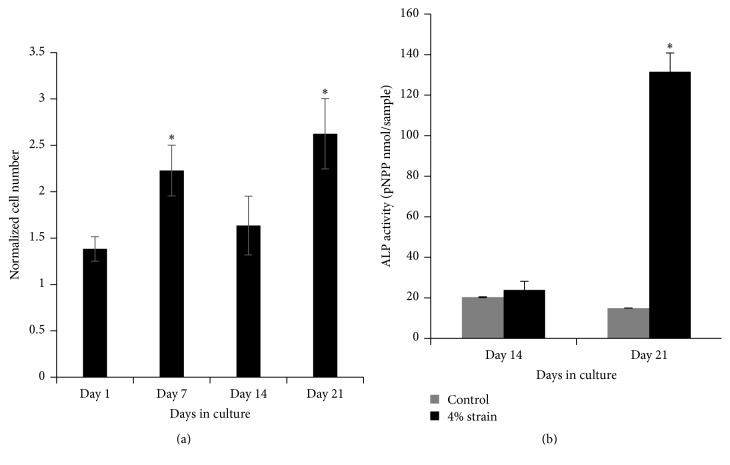
(a) Normalized MC3T3-E1 cell number within 4% mechanically strained collagen over 21 days. Normalized cell number is the ratio of cell number in mechanical strain group over cell number in the control group on the same culture day. *∗* indicates significant difference from day 1 experimental group. (b) The changes in ALP activity of MC3T3-E1 cell with equiaxial mechanical strain. *∗* indicates significant difference from other groups.

To understand whether cell viability results following mechanical loading application with EQUicycler data is cell-specific and whether the cell viability can be maintained for a longer culture period under mechanical loading, we have applied 4% equiaxial mechanical strain on osteoprogenitor cells (MC3T3-E1) for 21 days using EQUicycler.  Figure 7(a) demonstrates the changes in normalized MC3T3-E1 cell number over 21 days of mechanical loading.  Figure 7(a) also demonstrates that EQUicycler did not decrease the cell viability. In fact, normalized cell number (based on cell number on control) increased almost twofold on day 7 compared to day 1. On day 14, there is a slight decrease in cell number, which indicated the initiation of differentiation of the osteoprogenitor cells to osteoblasts. We have examined this fact using alkaline phosphatase (ALP) activity, early differentiation marker for osteoblast cells, for strained and control cells.  Figure 7(b) shows that mechanically strained cells using EQUicycler had higher ALP activity on day 21 compared to control group.

**Figure 8:**
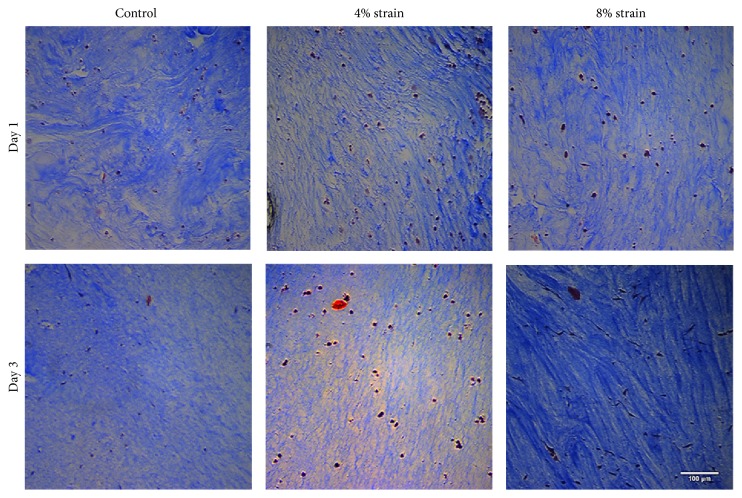
Histological evaluation of cell-embedded collagen constructs upon mechanical loading using EQUicycler. Masson's Trichrome was used to analyze the collagen fibers. Scale bar indicates 100 *μ*m.

*Collagen Matrix Morphology and Cell Morphology.* We further evaluated the morphological changes within the cell-embedded collagen matrix upon applying mechanical loading using EQUicycler for skeletal muscle cells (C2C12) and osteoprogenitor cells (MC3T3-E1).  Figure 8 demonstrates C2C12 cells-embedded collagen matrix prior to mechanical loading and after mechanical loading with EQUicycler. Differences in collagen matrix's fibrillar structure, their orientation, and morphology of residing cells over three days for control (unstrained), 4%, and 8% strained C2C12 cell-embedded collagen matrix were visualized using Mason's Trichrome stained histological samples. There was no physical damage or breakage within the matrix in response to the loading with EQUicycler over the three days of loading. On day 1, collagen matrix for control samples demonstrates more undulate fibrillar structure than equiaxial strained counterparts at the same day. For 4% and 8% strained constructs, the collagen matrix started to align on day 1 and continued to align over three days of mechanical loading. On day 3, collagen fibers within both 4% and 8% strained constructs demonstrate higher alignment compared to counterparts on day 1 for the same groups. Further, on day 3, while there was no change in collagen fiber thickness for control and 4% strained groups, 8% strained samples demonstrated thicker collagen fibers. The cells also aligned and showed elongated morphology with the collagen fibers for 8% strained construct while for 4% and control groups cells preserved their round morphology.

**Figure 9:**
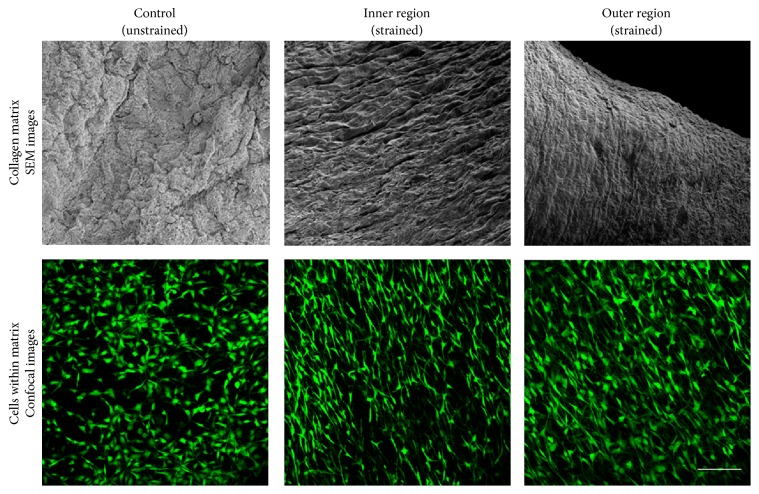
SEM images of the collagen matrix and confocal images of cells within the collagen matrix for control (unstrained) group and inner and outer region of mechanically strained group. The inner region is the inside of the collagen construct contacting with silicone post. Outer region is the edge of the collagen construct. The scale bar represents 100 *μ*m.

Applying mechanical strains on C2C12 cells using EQUicycler increases collagen fiber alignment and thickness and cells alignment for as early as three days of loading. In order to understand whether the effect of mechanical loading with EQUicycler on collagen fiber alignment and thickness are cell-specific, we applied 4% equiaxial strain with 1 Hz frequency to MC3T3-E1 cell-embedded collagen construct for 21 days using EQUicycler.  Figure 9 shows the SEM micrograph of MC3T3-E1 cell-embedded collagen construct for control (unstrained) sample and inner and an outer region of mechanically strained samples. SEM image of control sample demonstrates random collagen fiber distribution with undulated morphology. Yet, collagen fibers are highly aligned with both inner and an outer region in mechanically strained sample.

The cytoskeleton of cells was tagged with Calcein-AM to visualize the changes in cells morphology prior to and after mechanical loading. Cells residing within the control samples demonstrate random distribution within the collagen construct with relatively round morphology. On the other hand, for the inner and outer region of mechanically strained collagen construct, cells aligned and demonstrated elongated morphology.

## 4. Discussion

One of the critical challenges in applying mechanical strain to 3D cell-embedded matrix is the application of homogenous mechanical strain throughout the matrix. In commercial and noncommercial mechanical loading platforms to secure the cellular constructs to the platform, usually clamping systems are used. These clamping systems are responsible for creating stress concentration around the vicinity of the clamping apparatus. Flexcell (USA) is one of the most recognized commercial mechanical loading platforms utilized for a wide range of applications from musculoskeletal studies [35–38]. In this system, the cell-embedded 3D collagen construct is clamped from both ends and placed on a flexible membrane, which moves up and down using a vacuum system. While noncommercial mechanical loading platforms demonstrate vast variety in terms of design, sample loading configuration, and mechanical loading modalities, they all use clamping or post system to secure the cellular constructs to the platform. For instance, Birla et al. [39] developed one of the earlier mechanical loading platforms to apply uniaxial strain to the bioengineered heart muscle constructs (BEHM). In this system, BEHM is attached to the pins from one end and sutured to the other end. Goodhart et al. [40] developed a mechanical loading platform to apply uniaxial strain to the polyethylene terephthalate (PET) scaffolds seeded with mouse fibroblast cells (NIH-3T3). In this system, a scaffold is fixed from one end and clamped to an actuator on the other end. LabVIEW program is used to control the actuator to apply mechanical strains. Neidlinger-Wilke et al. [41] created a mechanical loading platform to apply uniaxial cyclic strain to the intervertebral disk cells-embedded collagen scaffolds. The cell-encapsulated collagen was poured into a rectangle silicone dish with three silicone posts at the right and left side of the dish. When collagen polymerized, collagen gripped the silicone posts and anchored to the dish. The loading device stretched the silicone dish to create uniaxial loading. Heher et al. [42] prepared skeletal muscle cell-embedded, ring-shaped fibrin matrix and applied cyclic or static mechanical stimulation through magnetic force. In this system, the ring-shaped scaffolds were mounted onto the spool-hook system to apply mechanical strain. In aforementioned and many other commercial and noncommercial mechanical loading platforms, the studies assumed that cells are exposed to same mechanical strain throughout the construct. The possible effect of stress concentration around the clamping system on cellular function is generally ignored. During cellular characterization, cells residing around high-stress areas are processed with the entire cell population within the construct. Yet, inconsistency in the experienced stress or strain may further lead to an error in data interpretation and conclusion about the role of stress in cellular functions.

The innovative mechanical loading platform, EQUicycler, described in this study was developed to apply various mechanical strains to 3D cell-embedded matrix without creating the gripping effect. EQUicycler allows depositing cell-embedded hydrogel around a silicone post, which is placed on the moving plates. The displacement of the moving plate against the fixed plate causes compression of the silicone post, which creates a mechanical strain on cell-embedded collagen ring surrounding the post (Figure 1). This way, in contrast to other mechanical loading platforms, EQ system does not require any apparatus including clamps, posts, and hooks to mount the matrix and to apply mechanical strains to the cell-embedded matrix. Thus, there is no inhomogeneous stress or strain concentration within the 3D cell-embedded construct when using EQUicycler system.

In order to confirm this fact, FEA model was built to plot strain contour for silicone post and surrounding collagen matrix around it (Figures 3 and 4). The representative strain contour for collagen (Figure 4(b)) demonstrated that there is no stress concentration within the collagen matrix in circumferential or longitudinal directions. The strain is homogenously distrusted in these directions. Further, through FEA model, five physiologically relevant strain values were applied, and strain gradients across the radial and longitudinal directions were plotted (Figures 5(a) and 5(b)). The changes in circumferential strain data with the various longitudinal direction (*Z* = 0, 0.25, 0.75, 1) demonstrated that circumferential strain is constant in the longitudinal direction for any given radial coordinates except *Z* = 0 location. With increased *Z* values (longitudinal position), the circumferential strain was constant for any given radial coordinates. The result seen for *Z* = 0 can be attributed to the deformation at the intersection of silicone post protrusion and silicone post base during loading. The deformation at this intersection was clearly depicted in strain contour images of silicone post (Figure 4(a)). FEA data confirmed that EQUicycler was effective in transferring equiaxial mechanical strain to the cell-collagen construct with minimal strain attenuation.

We have further investigated the performance of EQUicycler on cell viability and differentiation using relevant musculoskeletal tissue associated cells of skeletal muscle cells and osteoprogenitor cells. The skeletal muscle cells within collagen matrix were cultured using EQUicycler under tissue associated mechanical strain (4% and 8%), and cell viability was observed over three days of culture. The normalized cell number data (Figure 6) for skeletal muscle cells demonstrated that cell population increased with the increased mechanical strain and EQUicycler did not affect the viability of cells in a negative way. This data was further confirmed with osteoprogenitor cells. Prior to mechanical strain application, osteoprogenitor cells were not fully differentiated to osteoblasts yet. Thus, we have investigated the role of mechanical strain applied using EQUicycler on both cell viability and differentiation. Normalized cell number data for osteoprogenitor cells (Figure 7(a)) showed that cell viability was preserved over 21-day cell culture period, and overall cell number increased over time. The ALP activity, early osteogenic differentiation marker, also demonstrated that osteoprogenitor cells mechanically strained with EQUicycler showed a higher level of ALP activity compared to counterparts within the control samples. These data (Figures 6 and 7) proved that EQUicycler system did not decrease the cell viability and positively affected the osteoprogenitor cell differentiation towards osteoblasts. Although only two musculoskeletal related cell types have been utilized in this study to prove the performance of EQUicycler system, any type of adherent dependent cells can be mechanically strained using EQUicycler. Further, due to our laboratory's research interest, collagen was utilized to encapsulate the cells to create cellular construct. However, it should be noted that, in EQUicycler mechanical loading platform, there are no restrictions in matrix material choice and volume imposed by sample chamber or clamping system in contrast to other mechanical loading platforms. EQUicycler is compatible with many of the natural polymers used for fabricating hydrogel scaffolds for tissue engineering such as collagen, elastin, fibrin, Matrigel™, alginate, and chitosan.

EQUicycler was also very effective in aligning collagen matrix and residing cells within the matrix. The histology data (Figure 8) suggest that EQUicycler promoted active remodeling of the collagen with mechanical strain application. Mechanical loading applied using EQUicycler aligned collagen fibers increased the thickness of fibers and made cells elongate along with the collagen fibers within 3D construct. The matrix alignment was further confirmed by SEM images. The higher magnification images (Figure 9) of collagen in the control group and mechanically strained group demonstrated that EQUicycler was able to align collagen fibers located in both inner and an outer region of collagen construct compared to those in control groups. This data not only proves that EQUicycler promotes collagen fiber alignment but also proves that mechanical strain using EQUicycler was successfully transferred throughout the 3D collagen matrix from the inner region to the outer edge of the matrix. The morphology of cells (Figure 9) residing within the collagen constructs showed the similar trend. The cells aligned within the mechanically strained collagen construct in both inner and an outer region of the matrix, whereas cells within the control matrix demonstrated randomized arrangement. The alignment of the cell and their morphological changes upon mechanical strain can be attributed to the aligned collagen fibers. Our previous studies showed that cells clung onto collagen fibers when they were encapsulated within the collagen matrix [43]. Since fibrillar collagen has aligned with the mechanical loading, cells residing onto and in between these fibrillar structures have positioned themselves accordingly. Our histology and SEM images results that show aligned and more compacted collagen fibers for the strained samples agree with previous studies that reported that mechanical loading, as well as cell-mediated collagen gel compaction, resulted in aligned and thicker collagen fibers [44–47].

In sum, the EQUicycler system described in this study is a custom-made mechanical loading platform to create homogenous mechanical strain within the 3D cell-embedded hydrogel-based matrix. Its unique and cost-effective design make EQUicycler a powerful tool for mechanobiology studies.

## 5. Conclusion

This study describes the design, construction, and performance analysis of an innovative mechanical loading platform, EQUicycler. EQUicycler is effective in transferring equiaxial mechanical strain to the cell-collagen construct with minimal strain attenuation. The performance of EQUicycler was evaluated using Finite Element Analysis and experimental studies using various musculoskeletal tissue associated cell lines. The mechanical loading platform was proved to maintain and promote cell viability over extended periods of culture. Also, the early osteogenic differentiation marker of MC3T3-E1 cells, ALP, was upregulated due to mechanical stimulation performed using EQUicycler. Additionally, the matrix was actively remodeled due to mechanical strains. Collagen fibers were successfully aligned to the direction of the applied tensile equiaxial strain. Morphological changes were noticed for both C2C12 and MC3T3-E1 cells as they were also aligned and elongated in the direction of the applied strain.
